# Death Processes in Bovine Theca and Granulosa Cells Modelled and Analysed Using a Systems Biology Approach

**DOI:** 10.3390/ijms22094888

**Published:** 2021-05-05

**Authors:** Malgorzata J. McEvoy, Emilia Sinderewicz, Leo Creedon, Marion McAfee, Agnieszka W. Jonczyk, Katarzyna K. Piotrowska-Tomala, Dariusz J. Skarzynski

**Affiliations:** 1 Mathematical Modelling Research Group, Institute of Technology Sligo, Ash Lane, Sligo, F91 YW50 Sligo, Ireland; m.mcevoy@pan.olsztyn.pl (M.J.M.); mcafee.marion@itsligo.ie (M.M.); 2 Department of Reproductive Immunology and Pathology, Institute of Animal Reproduction and Food Research, Polish Academy of Sciences, Tuwima 10, 10-748 Olsztyn, Poland; a.jonczyk@pan.olsztyn.pl (A.W.J.); k.piotrowska-tomala@pan.olsztyn.pl (K.K.P.-T.); 3 Laboratory of Regenerative Medicine, Department of Neurosurgery, School of Medicine, University of Warmia and Mazury, 10-082 Olsztyn, Poland; emilia.sinderewicz@uwm.edu.pl

**Keywords:** systems biology, atresia, necroptosis, apoptosis, cluepedia, theca, granulosa, follicle, bovine reproduction

## Abstract

In this paper, newly discovered mechanisms of atresia and cell death processes in bovine ovarian follicles are investigated. For this purpose the mRNA expression of receptor interacting protein kinases 1 and 3 (*RIPK1* and *RIPK3*) of the granulosa and theca cells derived from healthy and atretic follicles are studied. The follicles were assigned as either healthy or atretic based on the estradiol to progesterone ratio. A statistically significant difference was recorded for the mRNA expression of a *RIPK1* and *RIPK3* between granulosa cells from healthy and atretic follicles. To further investigate this result a systems biology approach was used. The genes playing roles in necroptosis, apoptosis and atresia were chosen and a network was created based on human genes annotated by the IMEx database in Cytoscape to identify hubs and bottle-necks. Moreover, correlation networks were built in the Cluepedia plug-in. The networks were created separately for terms describing apoptosis and programmed cell death. We demonstrate that necroptosis (RIPK—dependent cell death pathway) is an alternative mechanism responsible for death of bovine granulosa and theca cells. We conclude that both apoptosis and necroptosis occur in the granulosa cells of dominant follicles undergoing luteinisation and in the theca cells from newly selected follicles.

## 1. Introduction

There are similarities in the mechanisms that control human and cattle reproduction. Therefore, models developed for cattle can provide information about processes taking place in humans [1,2]. The preovulatory follicle provides appropriate conditions for oocyte growth and development [3]. The ovarian follicle includes two main types of cells: granulosa and theca, which are responsible for nurturing the growing oocyte until ovulation. In cattle, the ovulating dominant follicle is recruited from 2 or 3 follicular waves [4], whereas the rest of the follicles growing in this same cohort undergo atresia [5,6,7]. It was estimated that 95–99% of all ovarian follicles in mammals undergo this process [8,9]. Atresia may occur at any time during folliculogenesis, however, the majority of follicles remain atretic throughout the antral stage of follicular development [6].

The studies concerning mechanisms of programmed cell death underlying atresia documented complex network of factors controlling this process [9,10]. However, the majority of investigations refer to apoptosis as the main mechanism regulating atresia. Apoptosis occurs via two main pathways: intrinsic and extrinsic. In the intrinsic pathway, the presence of pro-apoptotic factors or the absence of factors suppressing death, cause the changes in the inner membrane of the mitochondria and release of pro-apoptotic proteins from the intermembrane space into the cytosol [11]. Activation of these proteins lead to apoptosome formation and caspase (CASP9) activation, which initiates the executive CASP3 [12,13]. The extrinsic pathway depends on the transmembrane receptor-mediated interactions, belonging to the tumor necrosis factor (TNF) receptor gene superfamily (TNFRSF) [14]. The extracellular signals, such as Fas ligand (FasL) or tumor necrosis factor alpha (TNF-α), interact with corresponding death receptors [13,15,16]. In consequence, CASP8 initiates the activation of effector caspase cascade (e.g., CASP3, CASP7), resulting in the DNA fragmentation and apoptosis [12,17]. Granulosa cells are suggested as the initiator of follicular atresia, undergoing apoptosis in atretic follicles before the oocyte and theca cells [18,19,20]. However, it was proven that theca cells control granulosa cell apoptosis [21]. A study of Tajima et al. (2002) showed that paracrine factors produced by theca cells may inhibit apoptosis of granulosa cells [22].

Apoptosis in follicular atresia is controlled by a network of factors. Ovarian steroids, produced by granulosa and theca cells as a result of action by follicle-stimulating hormone (FSH) and luteinizing hormone (LH), are considered as a basis of this process. However, there are other regulators of follicular apoptosis. Kisspeptin, a stimulator of FSH and LH secretion, decreases viability of granulosa cells and initiates apoptosis in bovine granulosa cells [23]. Follistatin (FST), a local inhibitor of FSH secretion and insulin-like growth factor (IGF) family members, protects granulosa cells from apoptosis [24,25]. Furthermore, members of transforming growth factor beta (TGF-β) and TNF-α families were suggested as engaged in atresia regulation in cattle [26]. The latest studies show that also microRNA (miRNA) affects follicular atresia [9,27].

The latest investigations show necroptosis (in addition to apoptosis) as a mechanism controlling homeostasis in ovarian follicles. Necroptosis is an alternative form of programmed cell death, independent of CASPase [23]. Necroptosis is activated in response to factors promoting programmed cell death when the activation of caspases is blocked by their inhibitors. After recruitment of receptor interacting protein kinases (RIPKs) by death receptors, RIPK1, RIPK3 and mixed lineage kinase domain-like protein (MLKL) form a death-inducing signaling complex II. Phosphorylation of RIPK1, RIPK3 and MLKL results in cell death [28,29]. It is documented that spontaneous necroptosis occurs during luteolysis in primates and domestic animals [12,28,30]. The expression of necrosome components: RIPK1, RIPK3, MLKL and specific necroptosis marker phosphorylated MLKL (pMLKL(S358)), was documented in human and macaque ovarian follicles [13,31,32]. In cultured human granulosa cells, the presence of RIPK1 inhibitor and the MLKL blocker significantly reduced necroptosis [31]. It was also documented that the presence of the RIPK1 inhibitor resulted in increased diameters of secondary follicles and well-developed granulosa layers [32].

Although it is well known that apoptosis takes place during folliculogenesis [10], the role of necroptosis (programmed necrosis) dependent on RIPKs in this process is still open. There are few studies of the death processes in theca cells. Therefore the aim of this study was to investigate the occurrence or absence of necroptosis in the granulosa and theca cells of bovine ovarian follicles. For this purpose: (i) the mRNA expression of *RIPK1* and *RIPK3* of the granulosa and theca cells derived from the healthy and atretic follicles were studied by Real-Time PCR, and (ii) correlation between genes associated with death processes of the follicles at three different stages (newly selected, undergoing differentiation and undergoing luteinisation) was analysed by a systems biology approach.

A Systems Biology approach was taken to identify the differences/similarities between two types of cells (granulosa and theca cells) during two processes: apoptosis and programmed cell death. This motivated the creation of separate networks for granulosa and theca cells at different developmental stages. Cluepedia (an application available in Cytoscape) was used to create dynamic networks. These networks allowed the comparison of interactions between granulosa and theca cells and also permitted mapping of the known interactions (from the String-action database) on the newly created networks. Further, the DyNet Analyzer (another application available in Cytoscape) was used to visualise the similarities/differences between networks.

## 2. Results

### 2.1. mRNA Expression of RIPK1 and RIPK3 in Granulosa and Theca Cells Originating from Different Ovarian Follicle Types (Healthy and Atretic)

**Figure 1:**
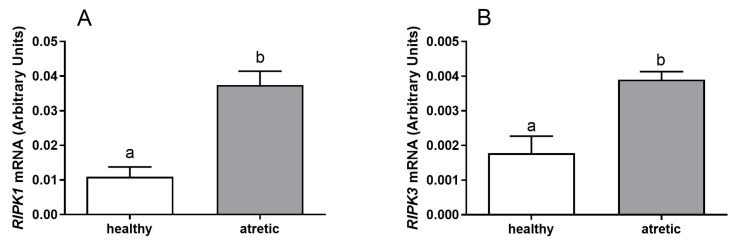
mRNA expression levels of (**A**) *RIPK1* and (**B**) *RIPK3* in granulosa cells of healthy and atretic follicles.

**Figure 2:**
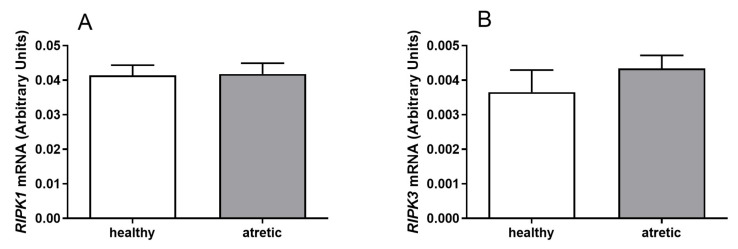
mRNA expression levels of (**A**) *RIPK1* and (**B**) *RIPK3* in theca cells of healthy and atretic follicles.

The mRNA expression of *RIPK1* and *RIPK3* was detected in granulosa cells (Figure 1) and theca cells (Figure 2) of healthy and atretic bovine ovarian follicles.

Greater mRNA levels of both *RIPK1* (Figure 1A, *p* < 0.05) and *RIPK3* (Figure 1B, *p* < 0.05) were found in the granulosa cells isolated from atretic follicles in comparison to healthy follicles. Similar mRNA levels of *RIPK1* (Figure 2A, *p* > 0.05) and *RIPK3* (Figure 2B, *p* > 0.05) were detected in theca cells isolated from both healthy and atretic follicle types. In summary, Figure 1 shows higher expression levels of *RIPK1* and *RIPK3* for granulosa cells from atretic follicles than for growing follicles. However, Figure 2 shows similar expression levels of *RIPK1* and *RIPK3* for theca cells from atretic and growing follicles.

### 2.2. The Protein-Protein Interaction Network

To study the interactome, the protein–protein interaction (PPI) network, based on the IMEx [33] curated database was created. Human data and Uniprot identifiers were used since interactions in mammals are conservative and the bovine interactions are scarcely annotated. Note that interactions found using IMEx [33] did not distinguish between interactions found in a specific tissue. Interactions between proteins allowed for study of the topological characteristics using MCODE (an application available in Cytoscape). This study allowed for the identification of the modules of genes associated with specific functions. The network was further enriched using the ClueGO application [19] and baseline expression of the chosen genes in the human ovary. The created network was used to study if there is experimental evidence for interactions between the proteins linked to necroptosis, apoptosis and atresia and to check how many of these proteins are expressed in the ovary. The chosen genes found in OMIM (Online Mendelian Inheritance in Man) [34] were mapped to the UniProtKB identifiers for humans.

The PPI network (protein–protein interaction) was created based on human protein interactions (the gene names and uniprot symbols used are shown in Appendix A Table A1). Functions played by proteins are closely conserved among species [16] and interactions recorded in humans are likely to occur similarly in bovines.

**Figure 3:**
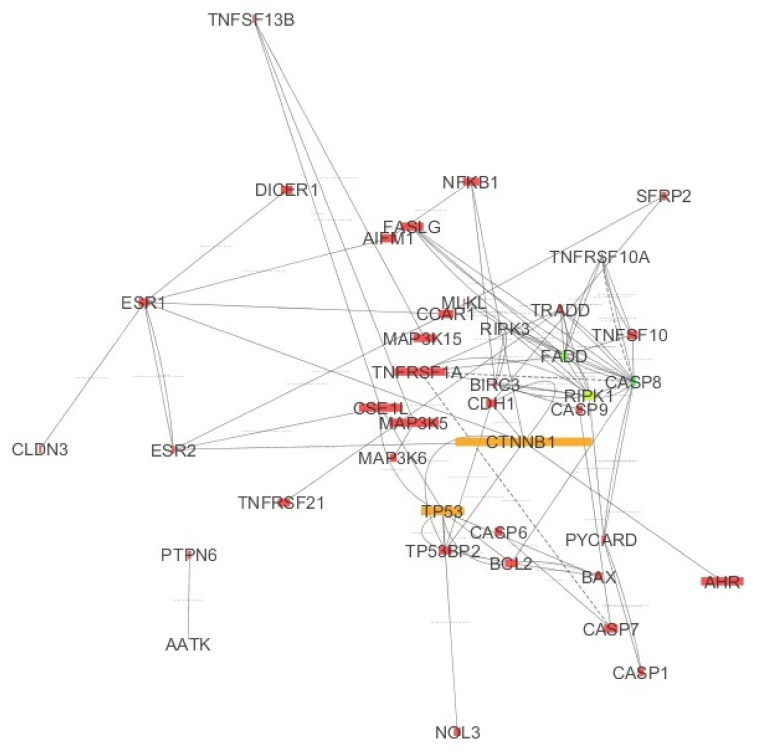
IMEx Network built based on the curated interactions available from the International Molecular Exchange (IMEx) consortium.

Moreover, the basal transcripts in the ovary found in the Gene Expression Atlas were integrated with the list of genes used to build the PPI network. The IMEx [33] network (only interactions in humans depicted) resulted in a network composed of 75 nodes and 297 edges. The PPI network contained many other proteins, which did not originate from the initial list. Thus, only the genes originating from the list of genes retrieved from OMIM [34] were selected (a network composed of 75 nodes and 297 edges). Next, a reduced network was created (with 75 nodes and 87 edges). This was done by removing self-interactions and multiple interactions between nodes. Next, nodes which did not interact with any other nodes were removed. This resulted in a network composed of 40 nodes and 87 edges. Finally, the topological characteristics were investigated using MCODE applied to the PPI network. The obtained IMEx [33] network is shown in Figure 3.

Figure 3 displays the IMEx Network [33], where the connections are not tissue specific. However, Figure 3 also shows the basal expression values of chosen genes in the ovary, mapped to the node size. As shown in Figure 3, *RIPK1* was expressed while *RIPK3* expression was very low (small node size). Note that *RIPK1* together with *FADD*, *TRADD*, *CASP8* and *TNFRSF1A* are expressed in apoptosis and they all occur in Figure 3. Thus, apoptosis may take place in the ovary. The complex: MLKL, RIPK3 and RIPK1 is characteristic of necroptosis. The direct connections between RIPK3 and MLKL and between RIPK1 and RIPK3 shown in the graph were derived from the IMEx database in which curated interactions (not tissue specific) are stored. The color of the nodes was mapped to the node degree (number of edges) (here green represents a high degree; yellow represents a medium degree; and red represents a low degree). Table A2 and Table A3 in the Appendix A show genes with the highest degree and betweenness centrality (the number of times a node acts as a bridge along the shortest path between two other nodes).

### 2.3. Network Clustering: Topological Clusters Using MCODE

The topological clusters found in MCODE identify groups of proteins with a similar function. The MCODE preprocessing combined the haircut option (dropping nodes from a cluster if there is only one connection to it) with default settings in CluePedia [19]. There were three topological clusters found in the IMEx network [33].

Cluster 1, containing: TRADD, FADD, CASP8, RIPK1 and TNFRSF1A (5 nodes and 16 edges).

Cluster 2, containing: TP53, BCL2 and BAX (3 nodes and 5 edges).

Cluster 3, containing: ESR1, CCAR1 and ESR2 (3 nodes and 4 edges).

Genes in Cluster 1 are needed for apoptosis. BAX and BCL2 from Cluster 2 determine the cell fate (apoptosis or not).

#### 2.3.1. Enrichment Study

Enrichment analysis identifies the over- and under- represented terms in the population.

The PPI network was enriched in the ClueGO application [19] using annotations from Reactome Pathways for humans [35]. The genes’ transcript used to create the network based on Reactome Pathways ontology [35] is specified in Table A1 in Appendix A.

**Figure 4:**
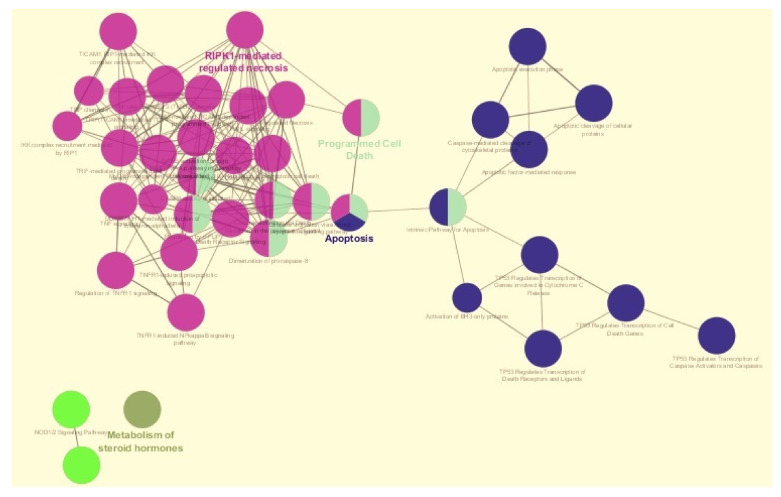
Enrichment results based on the Reactome pathways (17 February 2020) ontology.

The action relationships (expression, inhibition and activation) between the nodes were determined by CluePedia [19]. A file representing interactions between nodes was extracted from the STRING database [36] (on 17 February 2020) available in CluePedia [19]. Next, to assess functional annotation of the hub genes, ClueGO + CluePedia [19] were applied and biological pathways of the hubs in Figure 4 represent one or more functions (for example, the Apoptosis hub represents three functions). The pathways illustrated in Figure 4 which are associated with apoptosis and programmed cell death are studied in more detail using nested networks and CluePedia networks (see Section 5). The pathways are connected with the following settings in CluePedia [19]: assigned kappa score 0.5; corrected *p*-value < 0.05; number of genes per term equal to 3; percentage for the queried terms equals to 4; the Bonferroni setting was used with the *p*-value correction; and lastly two-sided (enrichment/depletion) tests based on hypergeometric distribution for terms and groups was selected. These settings were used also while retrieving functional annotations for the reduced list of genes. However, in the case of using the reduced list of genes, the Reactome Pathways database [35] was first used to create the functional terms.

#### 2.3.2. Analysis of GSE34317 Raw Data ($log_{2}$ Transformed Data for Granulosa and Theca Cells of Bovine Ovarian Follicles)

To study the death processes in the granulosa cells (GC) and theca cells (TC) of pre-ovulatory ovarian follicles the dataset GSE34317 was used (publicly available in NCBI’s Gene Expression Omnibus (GEO)) [37]. This is a dataset of gene expressions for 28,054 genes in the GC and TC of bovine dominant follicles at three different developmental stages.

“Principal component analysis (PCA) is a mathematical algorithm that reduces the dimensionality of the data while retaining most of the variation in the data set”. “It accomplishes this reduction by identifying directions, called principal components, along which the variation in the data is maximal. By using a few components, each sample can be represented by relatively few numbers instead of by values for thousands of variables. Samples can then be plotted, making it possible to visually assess similarities and differences between samples and determine whether samples can be grouped” [38].

**Figure A1:**
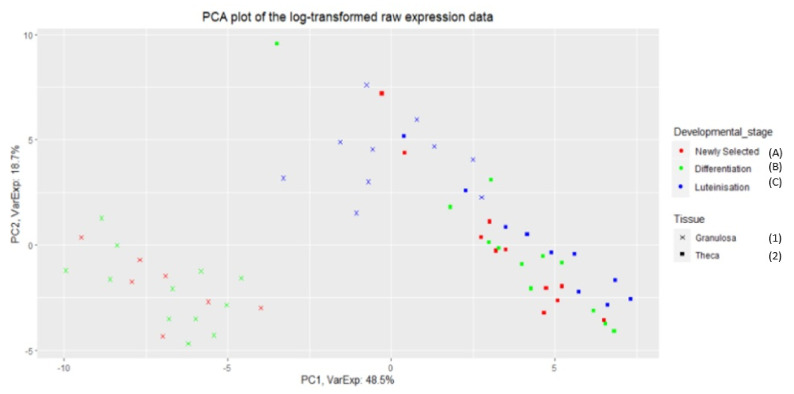
Principal Component Analysis (PCA) plot of $\log_{2}$ transformed raw data of GSE34317.

The PCA graph in Figure A1 shows the separation of data between two types of cells (granulosa and theca) of bovine ovarian follicles. In addition, the gene expressions (from Table A8 in Appendix A) of the granulosa cells differed between developmental stages. In contrast, expression of analysed genes was similar in the theca cells from all developmental stages. Moreover, the genes of the granulosa cells from the luteinising (atretic) follicles were more similar to theca than granulosa of healthy follicles (newly selected (A) or differentiating (B) stages).

Next, the down-regulated genes (in Table A4 in Appendix A) were identified in R using the DESeq2 package.

Tables (Table A4 and Table A5 in Appendix A) show that the smallest number of differentially expressed genes were in granulosa and theca cells originated from newly selected (A) and differentiating follicles (B). The expression of genes in granulosa cells of luteinising follicles differs from both granulosa and theca cells of newly selected and differentiating follicles. Note that *CYLD*, *RIPK1* and *CASP8* differed between group A1 vs. C1, and A1 vs. A2. *CYLD* and *RIPK1* differed between B1 vs. C1. *CYLD* differed between group A2 vs. C2. Moreover, *TNFRSF1A*, *RIPK1* and *TRADD* were down-regulated in granulosa compared to theca from newly selected follicles (group A1 vs. A2) and in granulosa from healthy follicles compared to those undergoing luteinisation (group A1 vs. C1 and B1 vs. C1). *RIPK3*, *RIPK1* and *CASP8* were down-regulated and *CYLD* was up-regulated in granulosa compared to the theca originated from follicles undergoing luteinisation. In theca cells originating from newly selected follicles compared to granulosa cells from follicles undergoing luteinisation (group A2 vs. C1), there was down-regulation of *CYLD* and up-regulation of *RIPK3* and *CASP8*. In addition, *CASP8* was up-regulated in group A1 vs. C1 and A1 vs. B1. When granulosa and theca were compared from the follicles undergoing luteinisation, down-regulation of *CASP8*, *RIPK3* and up-regulation of *CYLD* were recorded.

### 2.4. Nested Networks Describing Apoptosis and Programmed Cell Death

The apoptosis and programmed cell death gene networks shown in Figure 4 were analysed with nested networks. Nested networks were created and were composed of genes characteristic of either apoptosis or programmed cell death. Nested networks for apoptosis and programmed cell death were created based on the Reactome pathways [35] (see Figure 4). The connections between the genes in either apoptosis or programmed cell death were studied using String-action database and CluePedia networks. CluePedia [19] requires at least three replicates (samples) to conduct correlation analysis and create networks. Since these replicates were not available for theca of differentiating and luteinising follicles, CluePedia networks were created only for granulosa cells (all developmental stages) and theca cells of newly selected follicles. Only genes present in the original list (see Table A6 in Appendix A) were kept in the created networks (to improve readability). The activation and inhibition actions were found in “String” networks with String-Actions [36] (updated 7 September 2020). The action scores were set to 0.7 in CluePedia. In addition, the nested networks for apoptosis and programmed cell death were created and used to analyze experimental expression data from public repositories (GSE34317) using Pearson correlation and MIC (strength) algorithm available in CluePedia [19]. The Pearson correlation as well as MIC (strength) algorithm were set to 0.7 [19].

Separate networks were created for programmed cell death and apoptosis for two different types of cells (granulosa and theca). Three networks were created for granulosa cells, for follicles originating from growing, static and atretic follicles (the static follicles are the follicles undergoing differentiation).

The following genes occurred in the apoptosis pathway: *BAX, BCL2, CASP3, CASP6, CASP7, CDH1, CFLAR, FADD, FAS, PPP1R13B, RIPK1, TNFSF10, TP53, TRADD.* The following genes occurred in the programmed cell death pathway: *BAX, BCL2, BIRC3, CASP3, CASP6, CASP7, CASP8, CDH1, CFLAR, FADD, FAS, MLKL, PPP1R13B, RIPK1, RIPK3, TNFSF10, TP53, TRADD.*

**Figure 5:**
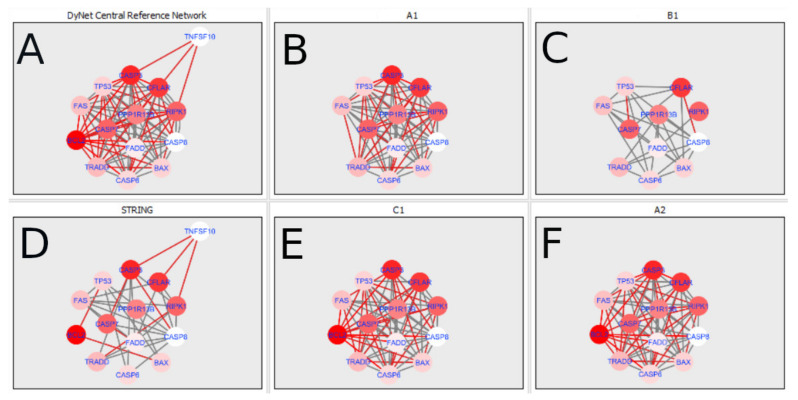
Apoptosis in granulosa and theca cells. The apoptosis network created based on the String-action database and the four networks created for apoptosis in both types of cells were used to build the DyNet Central Reference Network (shown in the bottom right image). The six networks (**A**–**F**), show interactions between genes). The genes which did not interact with other genes are not shown in this Figure, in order to improve readability. (**A**) shows Apoptosis DyNet Central Reference Network. (**B**) shows Apoptosis network created for group A1. (**C**) shows Apoptosis network created for group B1. (**D**) shows Apoptosis network created based on the String-action database. (**E**) shows Apoptosis network created for group C1. (**F**) shows Apoptosis network created for group A2. If the line is grey then that interaction is common to the six networks (otherwise it is red).

**Figure 6:**
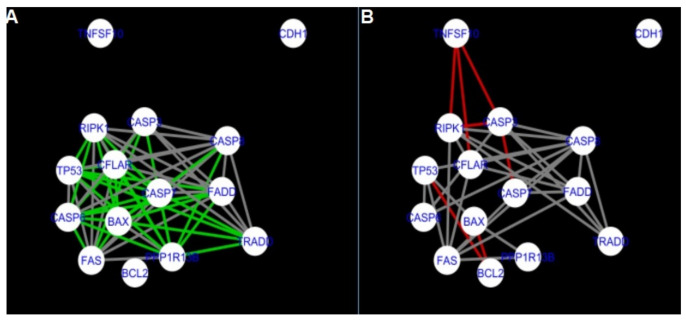
Apoptosis: Network created using String-action and in CluePedia for group A1. (**A**) shows the network built for granulosa cells of newly selected follicle (edges shown in green are characteristic for this network). (**B**) shows the network created based on the String-action interactions (edges marked in red are found only in the String-action network). Edges marked in grey occur in both networks.

**Figure 7:**
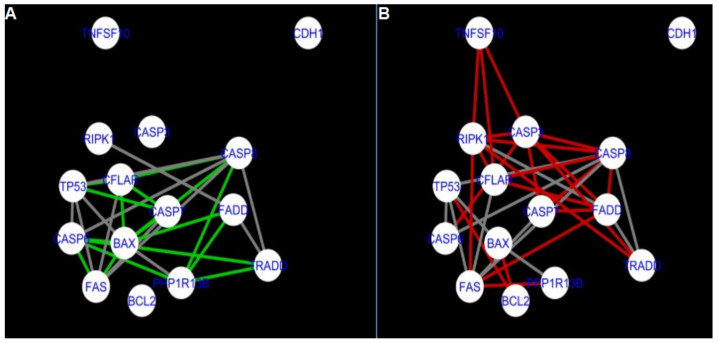
Apoptosis: Network created using String-action and in CluePedia for group B1. (**A**) shows the network built for granulosa cells of follicles undergoing differentiation (edges shown in green are characteristic for this network). (**B**) shows the network created based on the String-action interactions (edges marked in red are found only in the String-action network). Edges marked in grey occur in both networks.

**Figure 8:**
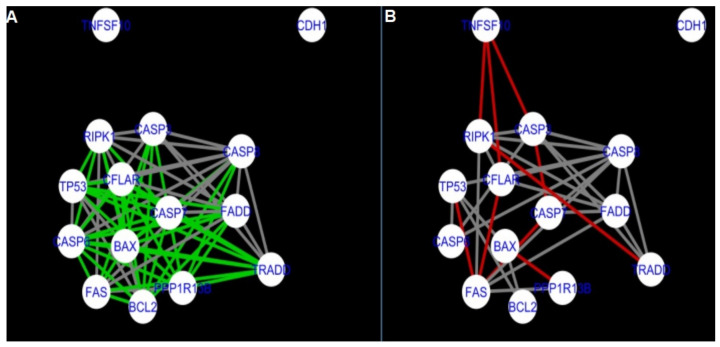
Apoptosis: Network created using String-action and in CluePedia for group C1. (**A**) shows the network built for granulosa cells of luteinising follicle (edges shown in green are characteristic for this network). In (**B**) is a network created based on the String-action interactions (edges marked in red are found only in the String-action network). Edges marked in grey occur in both networks.

**Figure 9:**
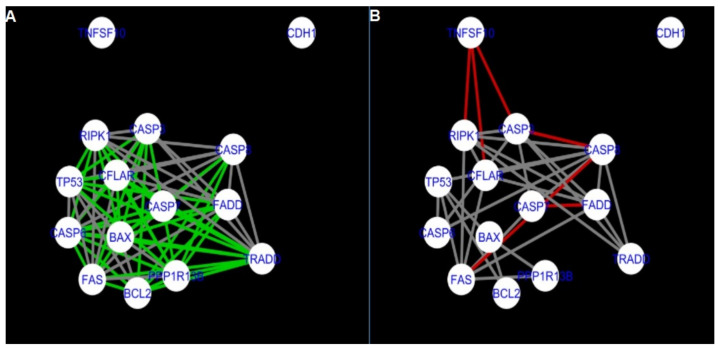
Apoptosis: Network created using String-action and in CluePedia for group A2. On the left (**A**) is the network built for theca cells of newly selected follicle (edges shown in green are characteristic for this network). On the right (**B**) is a network created based on the String-action interactions (edges marked in red are found only in the String-action network). Edges marked in grey occur in both networks.

**Figure 10:**
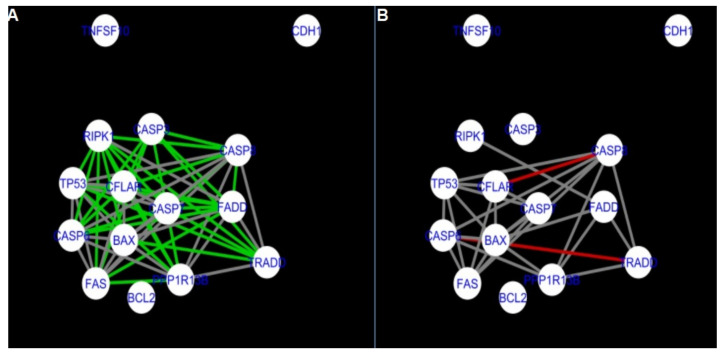
Apoptosis: CluePedia Network for group A1 vs. Cluepedia Network for group B1. On the left (**A**) is the network built for granulosa cells of newly selected follicles (edges shown in green are characteristic for this network). On the right (**B**) is a network created for granulosa cells of follicles undergoing differentiation (edges marked in red are found only in the Apoptosis network for group B1). Edges marked in grey occur in both networks.

**Figure 11:**
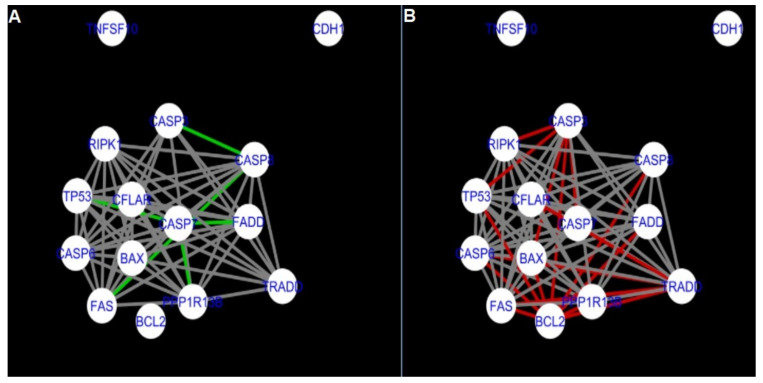
Apoptosis: CluePedia Network for group A1 vs. CluePedia Network for group A2. On the left (**A**) is the network built for granulosa cells of newly selected follicle (edges shown in green are characteristic for this network). On the right (**B**) is a network created for theca cells of newly selected follicles (edges marked in red are found only in the Apoptosis network for group A2). Edges marked in grey occur in both networks.

**Figure 12:**
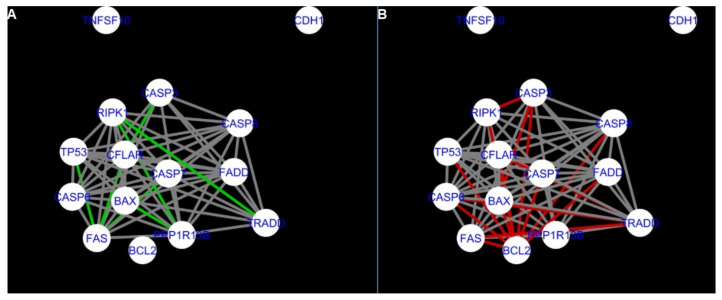
Apoptosis: CluePedia Network for group A1 vs. CluePedia Network for group C1. On the left (**A**) is the network built for granulosa cells of newly selected follicle (edges shown in green are characteristic for this network). On the right (**B**) is a network created for granulosa cells of luteinising follicles (edges marked in red are found only in the Apoptosis network for group C1). Edges marked in grey occur in both networks.

**Figure 13:**
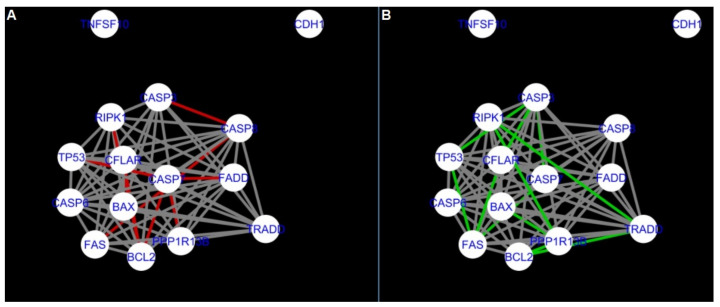
Apoptosis: CluePedia Network for group C1 vs. CluePedia Network for group A2. On the left (**A**) is the network built for granulosa cells of luteinising follicles (edges shown in green are characteristic for this network). On the right (**B**) is a network created for theca cells of newly selected follicles (edges marked in red are found only in the Apoptosis network for group A2). Edges marked in grey occur in both networks.

**Figure 14:**
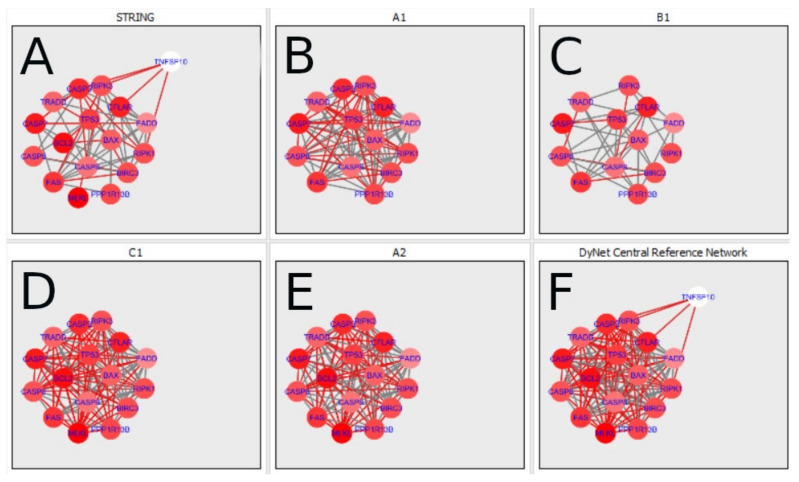
Programmed Cell Death in granulosa and theca cells. The Programmed Cell Death network created based on the String-action database and the four networks created for Programmed Cell Death in both types of cells were used to build the DyNet Central Reference Network (shown in the bottom right image). The six networks show interactions between genes. The genes which did not interact with other genes are not shown in this Figure, in order to improve readability. (**A**) shows Programmed Cell Death network created based on the String-action database. (**B**) shows Programmed Cell Death network created for group A1. (**C**) shows Programmed Cell Death network created for group B1. (**D**) shows Programmed Cell Death network created for group C1. (**E**) shows Programmed Cell Death network created for group A2. (**F**) shows Programmed Cell Death DyNet Central Reference Network. If the edge is grey then that interaction is common to the six networks (otherwise it is red).

**Figure 15:**
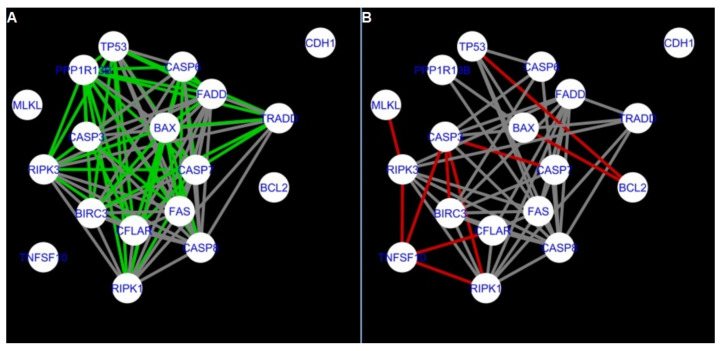
Comparison of CluePedia networks for Programmed Cell Death created using String action vs. Group A1. On the left (**A**) is shown network built for granulosa cells of newly selected follicle (edges shown in green are characteristic for this network). On the right (**B**) is a network created based on the String-action interactions (edges marked in red shown edges found only in the String-action network). Edges marked in grey occurs in both networks.

**Figure 16:**
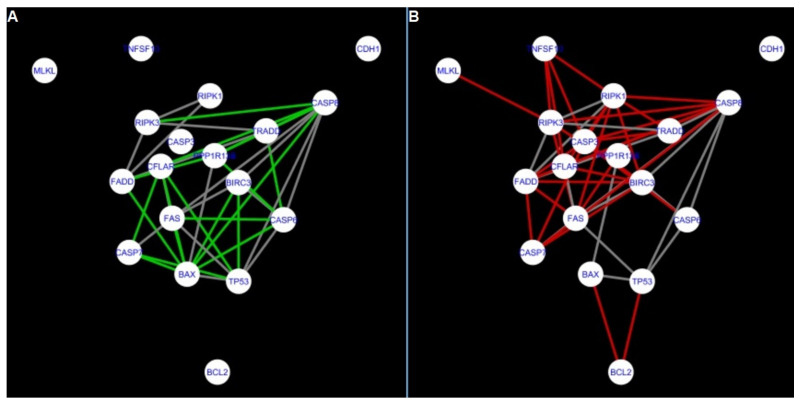
Comparison of CluePedia networks for Programmed Cell Death created using String action vs. Group B1. On the left (**A**) is the network built for granulosa cells of follicles undergoing differentiation (edges shown in green are characteristic for this network). On the right (**B**) is a network created based on the String-action interactions (edges marked in red are found only in the String-action network). Edges marked in grey occur in both networks.

**Figure 17:**
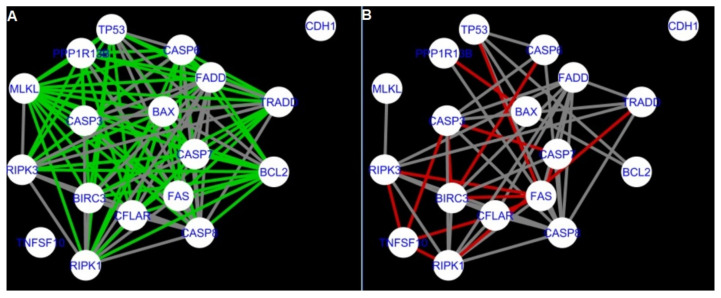
Comparison of CluePedia networks for Programmed Cell Death created using String action vs. Group C1. On the left (**A**) is the network built for granulosa cells of luteinising follicles (edges shown in green are characteristic for this network). On the right (**B**) is a network created based on the String-action interactions (edges marked in red are found only in the String-action network). Edges marked in grey occur in both networks.

**Figure 18:**
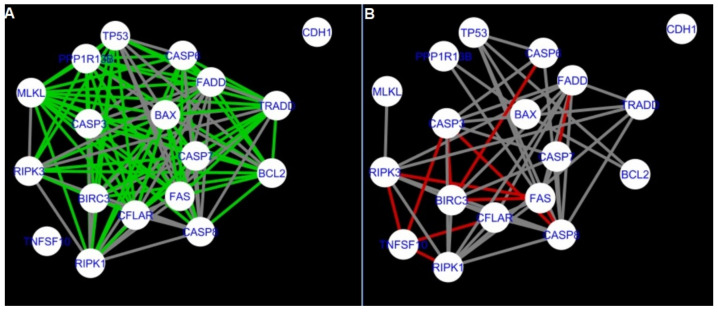
Comparison of CluePedia networks for Programmed Cell Death created using String action vs. Group A2. On the left (**A**) is a network built for theca cells of newly selected follicle (edges shown in green are characteristic for this network). On the right (**B**) is a network created based on the String-action interactions (edges marked in red are found only in the String-action network). Edges marked in grey occur in both networks.

**Figure 19:**
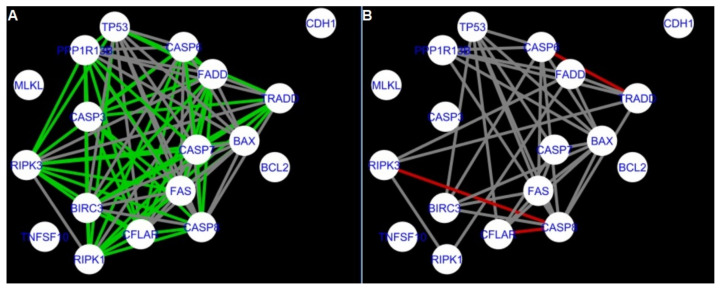
Comparison of CluePedia networks for Programmed Cell Death in Group A1 vs. Group B1. On the left (**A**) is a network built for granulosa cells of newly selected follicles (edges shown in green are characteristic for this network). On the right (**B**) is a network created for granulosa cells of follicles undergoing differentiation (edges marked in red are found only in the Programmed Cell Death network of group B1). Edges marked in grey occur in both networks.

**Figure 20:**
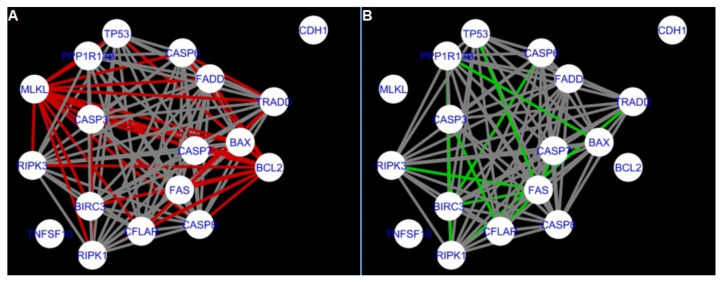
Comparison of Cluepedia networks for Programmed Cell Death in Group A1 vs. Group C1. On the left (**A**) is a network built for granulosa cells of newly selected follicle (edges shown in green are characteristic for this network). On the right (**B**) is a network created for granulosa cells of luteinising follicles (edges marked in red are found only in the Programmed Cell Death network of group C1). Edges marked in grey occur in both networks.

**Figure 21:**
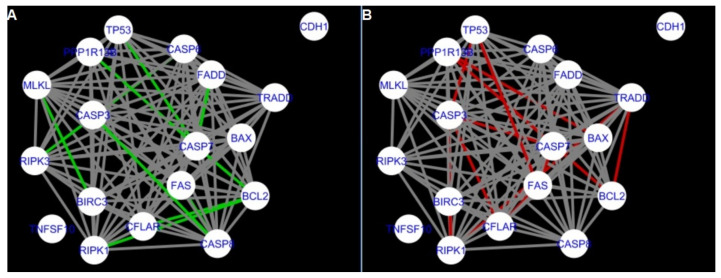
Comparison of CluePedia networks for Programmed Cell Death in Group A2 (theca) vs. Group C1. On the left (**A**) is a network built for theca cells of newly selected follicle (edges shown in green are characteristic for this network). On the right (**B**) is a network created for granulosa cells of luteinising follicles (edges marked in red are found only in this network). Edges marked in grey occur in both networks.

The DyNet Analyzer application available in Cytoscape allowed the visual comparison between networks with ‘known’ interactions (‘String networks’) and newly created networks in CluePedia (based on the results from the GSE34317 database). Note that data used to create CluePedia networks were rma normalised (and were described in Section 4.4.2). The DyNet Analyzer highlighted the most ‘rewired’ nodes [39] in CluePedia networks (more strongly connected to different neighbours in different networks). Figure 5, Figure 6, Figure 7, Figure 8, Figure 9, Figure 10, Figure 11, Figure 12 and Figure 13 refer to the process of apoptosis and Figure 14, Figure 15, Figure 16, Figure 17, Figure 18, Figure 19, Figure 20 and Figure 21 to the process of programmed cell death. Figure 5 and Figure 14 show differences/similarities between networks created for different cells at different developmental stages and the networks created based on the known interactions (using String-action), respectively, for apoptosis and programmed cell death. In addition, a reference network containing the union of all network states was generated.

To further improve the readability of the networks, they were compared one at a time to a network built based on the known non-tissue specific interactions (‘String’ networks) (Figure 6, Figure 7, Figure 8 and Figure 9 and Figure 15, Figure 16, Figure 17 and Figure 18).

In addition, networks built based on the experimental data were compared to each other as shown in Figure 10, Figure 11, Figure 12 and Figure 13 for apoptosis and in Figure 19, Figure 20 and Figure 21 for programmed cell death. Throughout the paper, necroptosis is used synonymously for programmed cell death.

Firstly, the nested networks of apoptosis created for the tested groups were compared to the nested network of apoptosis, in which connections were created based on the String-action database. Recall that the interactions stored in the String-database are derived from many different human tissues. Thus, it is expected that the interactions will differ to some extent from networks created for granulosa and theca cells originating from bovine ovarian follicles.

Next, the networks for groups A1, B1, C1 and A2 were compared to each other. A similar process was repeated for the nested networks of programmed cell death. In each case, edges in one network were coloured red, while edges in the other network were coloured green. Edges present in both networks were coloured grey.

Figure 5 shows created networks of apoptosis for granulosa cells originating from all developmental stages of follicles, from theca cells of newly selected follicles, the network created based on the String-action interactions, and also the DyNet Central Reference Network built based on all of them.

The nested networks for programmed cell death are shown in Figure 14, Figure 15, Figure 16, Figure 17, Figure 18, Figure 19, Figure 20 and Figure 21.

Figure 14 shows the created networks of programmed cell death for granulosa cells originating from all developmental stages of follicles, for theca cells of newly selected follicles, the network created based on the String-action interactions and also the DyNet Central Reference Network built based on all of them.

The comparison of the networks shows that the most similar were nested networks of apoptosis as well as programmed cell death created for granulosa cells of luteinising follicles (C1) and theca cells of newly selected follicles (A2).

In the two groups (C1 and A2) direct connections between *RIPK1*, *RIPK3* and *MLKL* were visualised in the networks of programmed cell death (Figure 21). In the created networks the lack of direct connection between *BAX* and *BCL2* in granulosa cells of newly selected follicles as well as follicles undergoing differentiation was also depicted.

The list of genes used to create the CluePedia networks for respective groups is shown in Table A6 in Appendix A. There was missing expression data for *MLKL* in the granulosa cells (in group A1 and B1 there was only one *MLKL* reading available). There was also missing data for *STK17A* in group B1 and A1; and for *NAIP* in group A1.

## 3. Discussion

The literature describes the use of animal models to study the reproductive processes occurring in humans [1,2]. In the presented work, we used databases on both cows and humans to expand our understanding of death processes. It should be noted that the results and conclusions expressed for dominant follicles undergoing luteinization are extrapolations from human databases. The results of this paper provide new information on the mechanisms involved in the death processes of ovarian follicles in cattle, a species that can serve as a model for studying reproductive processes in other animal species, including humans. Recently, Sinderewicz et al. [40] described apoptosis of bovine granulosa cells. However, our study completes the knowledge of the death process in bovine ovarian follicles, demonstrating that bovine granulosa and theca cells are eliminated not only by apoptosis but also by programmed necrosis (RIPK-dependent necroptosis). In our study, the mRNA expression of the main factors related to necroptosis in bovine granulosa and theca cells isolated from both healthy and atretic follicle were detected: *RIPK1* and *RIPK3*. Moreover, based on the systems biology approach, the death processes in the granulosa and theca cells of the dominant follicles were investigated. The nested networks technique was used based on MIC (strength) and Pearson correlation set to r = 0.7 in CluePedia. The nested networks were created for two terms: for programmed cell death and for apoptosis. CluePedia [19] requires at least three replicates (samples) to conduct correlation analysis and create networks. Since these replicates were not available for theca of differentiating and luteinising follicles, CluePedia networks were created only for granulosa cells (at all developmental stages) and theca cells of newly selected follicles.

Studies have shown that the apoptotic process occurs in the early- and middle-stage bovine corpus luteum (CL) [41] and in bovine granulosa cells [40]. Sinderewicz et al. [40] demonstrated an increase in factors involved in cell apoptosis, e.g., CASP8 of the atretic follicles compared to the healthy follicles. Moreover, our study of DEG based on the GSE34317 experimental data confirm that there is an increase in *CASP8* in granulosa cells from luteinizing follicles and in theca cells from newly selected follicles. It is well known that an activated CASP8 is required for cleaving the CASP3 promotor of the apoptotic pathway [42]. In our study, *CASP3* was upregulated in group A1 vs. group A2 and in group A1 vs. group C1 (GSE34317 data). However, the interaction between CASP3 and CASP8 was missing in the apoptosis network in the granulosa cells undergoing differentiation. We described that in group B1, the FADD was needed for formation of the RIPK1, TRADD and CASP8 complex (thanks to the direct connection between FADD and RIPK1; between FADD and TRADD; and between TRADD and CASP8). No direct link was found between RIPK1 and TRADD in the granulosa cells of luteinising follicles, indicating that death processes are more complex in follicles undergoing luteinisation. It is important to know that RIPK1 combines with FADD, TRADD, RIPK3 and CASP8 to form complex II [43]. Active CASP8 promotes the exogenous apoptosis pathway by inactivating RIPK1 and RIPK3 [44]. The above interactions were found in our experimental data (GSE34317) based on gene expression and correlation networks. *RIPK1*, *CASP8* and *TRADD* were downregulated in group B1 vs. C1; A1 vs. C1; and A1 vs. A2. In contrast *RIPK1*, *CASP8* and *TRADD* were upregulated in group A2 vs. C1. Interactions between *RIPK1* and *CASP8*; *CASP8* and *TRADD*; and *TRADD* and *RIPK1* were found in the nested networks of apoptosis created for group A1. However, in granulosa cells undergoing differentiation, the complex consisting of *RIPK1*, *CASP8*, *TRADD* and *FADD* was found in the nested network of Apoptosis. Note that in group B1 and group C1, no direct interaction between *RIPK1* and *TRADD* was found. Thus, in the nested network for apoptosis, a connection between *RIPK1*, *TRADD*, *FADD* and *CASP8* was found in the granulosa cells at all three developmental states and in the theca cells of newly selected follicles. These four genes are characteristic of apoptosis ongoing in the CL and indicate that apoptosis also takes place in the bovine granulosa cells (at all developmental stages) and theca cells originating from newly selected follicles. Thus, our results clearly describe that apoptosis takes place in granulosa and theca cells.

Recently, necroptosis was found in human granulosa cells [45] and played an important role in the death of bovine CL [28]. Zhang et al. [46] found that during embryonic development RIPK1 can mediate apoptosis as well as necroptosis. Moreover, for successful embryogenesis FADD is needed (FADD suppresses RIPK1 and RIPK3 dependent necroptosis) [46]. The differences in the expression of *RIPK1* and *RIPK3* between granulosa and theca of newly selected follicles may be due to the role of theca cells in suppressing apoptosis of the granulosa cells [22]. In our results a difference was recorded in the mRNA expression of *RIPK1* and *RIPK3* between granulosa cells originated from the healthy and atretic follicles. Moreover, nested networks created for programmed cell death show a direct interaction between RIPK1 and RIPK3 in granulosa cells originated from bovine follicles of all developmental stages. Both RIPK1 and RIPK3 are needed for apoptosis, however low levels of RIPK1 and high levels of RIPK3 induce necroptosis [47]. In the study of Walsh et al. [48] (GSE34317), data they have recorded upregulation of *RIPK3* in group A2 vs. group C1. *RIPK3* has an anti-viral effect promoting cytokine gene expression [49]. For instance, this occurs in the case of vaccinia virus infection during which RIPK3 dependent necroptosis mobilises immune cells against the virus [50]. Jonczyk et al. [29] confirmed that not only RIPKs, but also CYLD and MLKL are involved in the necroptotic pathway during prostaglandin F2α-induced CL regression. During necroptosis, CYLD causes deubiquitination of RIPKs because of recruitment of RIPKs by death receptors [50]. In our study, *CYLD* was downregulated in theca and granulosa cells from newly selected follicles compared to the theca and granulosa originating from luteinising follicles. *CYLD* was upregulated in granulosa compared to theca cells of luteinising follicles (C1 vs. C2 and A1 vs. A2). In these two groups (C1 and C2) there was also a recorded difference in expression of *RIPK3* and *CASP8* (which were downregulated in group C1 vs. C2). Thus, the above results show that the necroptosis process takes place in both the granulosa and theca cells of the dominant follicle undergoing luteinisation. The phosphorylation of RIPK3 is crucial for the recruitment of MLKL [13,51] and is characteristic of necroptosis. In our study, the complex: *RIPK1*, *RIPK3* and *MLKL* was found in the nested network of programmed cell death for granulosa cells originating from follicles undergoing luteinisation (C1), and theca cells of newly selected follicles (A2). The similarity between expression of genes involved in death processes in granulosa cells of the luteinizing follicles and theca cells at all three developmental stages are also visualised on the PCA graph (see Figure A1). In the nested network a direct connection between *RIPK3* and *MLKL* was found in the theca cells originated from newly selected bovine follicles, and in granulosa cells of follicles undergoing luteinisation. The DEG results confirm *MLKL* and *RIPK3* up-regulation in the theca of newly selected follicles compared to the theca from the follicles undergoing differentiation (based on the experimental data taken from the GEO database (as shown in Table A5 in Appendix A)). In contrast, in theca cells undergoing differentiation MLKL was downregulated compared to theca from luteinising follicles. Note that the experimental study did not show a difference between the expression of *RIPK1* and *RIPK3* in the theca cells originated from the healthy and atretic follicles. This difference was also not recorded in theca cells from newly selected and luteinising follicles.

There are studies confirming occurrence of both apoptosis as well as necroptosis in the granulosa cells [40,45]. To our knowledge, this paper demonstrates the occurrence of necroptosis in the bovine theca cells for the first time. This information might be used to implement necroptosis inhibitors allowing for manipulation of the death processes, this can have practical applications in reproduction programs. Necroptosis is associated with inflammatory diseases [52]. TNFα-induced necroptosis promotes the expression of proinflammatory cytokines [53]. Hou et al. [52] show that PK68 can be used for targeting RIPK1, acting as a RIPK1 inhibitor. Furthermore, research by Zhu et al. [53] found that induction of cytokine by necroptosis can be inhibited by targeting RIPK1 (using Nec-1s); RIPK3 (using GSK872) and inhibiting MLKL oligomerization using necrosulfonamide. In contrast, necroptosis can be promoted by TAM (Tyro3, Axl, and Mer) family of tyrosine kinases receptor, pharmacological or genetic targeting which results in inhibition of necroptotic death [49]. Necroptosis can be blocked by necrostatin-1 (Nec-1) or derivatives, and the MLKL inhibitor necrosulfonamide (NSA) [31]. Thus, pharmacological agents can be used to prolong the availability of the follicle pool during ageing (and in humans can possibly delay the menopause) [31]. This showed that ovarian function and fertility of primates can be improved by pharmacological intervention targeting specific death processes [31]. This is one of the reasons why identification of the mechanisms of cell death processes is important.

## 4. Materials and Methods

### 4.1. Experimental Methods

Ovaries, from dairy cows, irrespective of the stage of the estrous cycle, were collected at the slaughterhouse and transported to the laboratory in sterile Phosphate Buffered Saline within 30 min (PBS buffer (137 mM NaCl, 2.7 mM KCl, 10 mM Na2KPO4, 1.8 mM KH2PO4, pH = 7.4), completed with 0.5% gentamycin (Sigma-Aldrich, Steinheim, Germany; #G-1397)). Materials were collected throughout the whole year.

The follicular fluid (FF) was aspirated from antral ovarian follicles (diameter < 5 mm) by syringe. Then the antral cavity of each follicle was rinsed frequently with cold PBS to collect the granulosa cells. Granulosa cells were separated from the FF by centrifugation at 1200× *g* for 4 min. The theca layer was removed from the inner part of the follicles with forceps and washed in PBS by passing repeatedly through a 1 mL syringe. Theca and granulosa cells obtained from single follicles were collected and stored at −80 ${}^{\circ }$C until the RNA extraction process. The supernatant of FF was collected and kept frozen at −20 ${}^{\circ }$C until the concentrations of E2 and P4 were measured by the RIA method (using the DIAsource E2–RIA–CT Kit, KIP0629, DIAsource ImmunoAssays SA, Louvain-la-Neuve, Belgium and the DIAsource PROG–RIA–CT Kit, KIP1458, Diasource, ImmunoAssays SA, Louvain-la-Neuve, Belgium).

Granulosa cells and theca layer were divided into two categories: healthy (E2:P4 ratios > 1) and atretic (E2:P4 ratios < 0.01) based on the intrafollicular E2:P4 ratios and used for *RIPK1* and *RIPK3* mRNA expression examination.

### 4.2. Total RNA Extraction, Reverse Transcription (RT) and Real-Time PCR

The samples were homogenized and processed for RNA isolation according to manufacturer’s instructions (#031-100, Total RNA Mini Isolation Kit, AA Biotechnology, Poland). Samples were stored at −80 ${}^{\circ }$C until RT. Before use, RNA content and purity were evaluated by spectrophotometric measurement (NanoDrop 1000 ; Thermo Scientific, Wilmington, DE, USA). RT was performed using oligo (dT) 12–18 primers (#18418-012) by Maxima First Strand cDNA Synthesis Kit (#K1642, Fermentas, Thermo Fisher Scientific, Wilmington, DE, USA) in a total volume of 20 L.

The expression of mRNA for *RIPK1* and *RIPK3* was measured by Real-time PCR. Real-time PCR was performed with an ABI Prism 7900 (Applied Biosystems, Life Technologies, Foster City, CA, USA) sequence detection system using Maxima^®^ SYBR Green/ROX qPCR Master Mix (# K0222, Fermentas, Thermo Scientific, Wilmington, DE, USA). The PCR reactions were performed in 384-well plates. The results of mRNA transcription were normalized to the glyceraldehyde-3-phosphate dehydrogenase (*GAPDH*, an internal control) mRNA level and were expressed as arbitrary units. The primers were designed using an online software package (http://frodo.wi.mit.edu/primer3/input.htm (accessed on 15 March 2018)). The primer sequences and the sizes of the amplified fragments of the transcripts are shown in Table A7 in Appendix A. For the relative quantification of mRNA levels, Miner software was used (http://www.miner.ewindup.info (accessed on 30 August 2018).

### 4.3. Estradiol and Progesterone Assays

Estradiol and P4 were measured by the radioimmunoassay method (DIAsource E2–RIA–CT Kit, KIP0629, DIAsource, ImmunoAssays SA, Louvain-la-Neuve,Belgium; PROG–RIA–CT Kit, KIP1458, Diasource, ImmunoAssays SA, Louvain-la-Neuve,Belgium) according to the manufacturer’s instructions.

### 4.4. Data Analysis and Statistics

#### 4.4.1. RT-PCR Data Analysis

The mRNA expression of *RIPK1* and *RIPK3* was measured for granulosa and theca cells of bovine follicles. Statistical analyses were performed using GraphPad PRISM v.6.0 software (GraphPad Software, San Diego, CA, USA). All experimental data are shown as the mean ± SEM, and differences were considered to be statistically different at the 95% confidence level (*p* < 0.05). Analyses were done using one-way ANOVA followed by Kruskal–Wallis’ multiple comparison test (Figure 1 and Figure 2).

#### 4.4.2. Systems Biology Methods

To study the death processes in the granulosa cells (GC) and theca cells (TC) of pre-ovulatory ovarian follicles the dataset GSE34317 was used (publicly available in NCBI’s Gene Expression Omnibus (GEO)) [37]. This is a dataset of gene expressions for 28,054 genes in the GC and TC of bovine dominant follicles at three different developmental stages. The chosen genes characterised as important for necrosis, apoptosis and atresia processes were identified using an online catalog of human genes and genetic disorders (OMIM) [34]. Next, the chosen genes were selected from the GSE34317, followed by rma normalisation. Due to the lack of the annotation file, rma normalisation was performed in three steps: background correction using rma.background.correction function in R; followed by quantile normalisation using normalize.quantiles function in R; and logarithmic transformation using convert_log2 function in R [54]. These functions are available in R packages preprocessCore and proteoMM. Due to the limited number of samples for which rma normalisation was successful, the resulting dataset contained the following smaller sample: GSE847251 (GCA), GSE847254 (GCA), GSE847256 (GHA), GSE847263 (GCB), GSE847264 (GCB), GSE847265 (GCB), GSE847266 (GCB), GSE847267 (GCB), GSE847279 (GHC), GSE847280 (GHC), GSE847282 (GHC), GSE847284 (TCA), GSE847286 (TCA), GSE847290 (THA). For instance, GSE847251 (GCA) means Granulosa Cow Group A (newly selected), sample identifier GSE847251; and THB stands for Theca Heifer Group B. Samples contained expression data recorded in either cows or heifers in granulosa and theca cells originated from one of the 3 developmental stages of follicles:

A (newly selected), B (undergoing differentiation) or C (undergoing luteinisation).

The data were divided into 6 groups (3 for granulosa and 3 for theca). The method in which samples were assigned to the specific groups is shown in Table 1.

$\log_{2}$ transformed raw data was used for Principal Component Analysis (PCA) and for analysing up- and down- regulated genes. PCA reduces the dimensionality of the dataset allowing for visualising similarities/dissimilarities of data using principal components of maximal variation [38].

In addition, the list of genes used for differentially expressed gene (DEG) analysis and PCA were limited (compared to the list of genes retrieved from OMIM and used for creation of an IMEx network) due to zero values for expression of some genes recorded in the dataset. These genes were removed from the analysis, except results for *RIPK3.* Zero values for *RIPK3* were recorded in 3 samples: GSE847252, GSE84725253 and GSE847255. In order to avoid an elimination of the results for *RIPK3*, the samples with no recorded expression for *RIPK3* were removed. The final gene transcripts in the dataset are shown in Table A8 in Appendix A.

## 5. Conclusions

Results of RT-PCR analyses showed statistical differences in the mRNA expressions of *RIPK1* and *RIPK3* in granulosa cells from healthy follicles and granulosa cells from atretic follicles but it was not observed in theca cells. The results of the system biology (networks) approach showed strong relationships between *RIPK1*, *RIPK3* and *MLKL* (characteristic of necroptosis) in the programmed cell death network for the granulosa cells of luteinising follicles. Connections between *MLKL*, *RIPK1* and *RIPK3* were also found in the nested network of programmed cell death in the theca cells of newly selected follicles. Thus, both apoptosis and necroptosis occurs in the granulosa cells of luteinising preovulatory follicles and in the theca cells of newly selected follicles. Moreover, the expression values of genes created two clusters on the PCA graph: one cluster containing data for granulosa (groups A1 and B1); and the other cluster for granulosa (group C1) and theca (groups A2, B2 and C2).

In conclusion, bovine granulosa and theca cells are eliminated not only by apoptosis but also by programmed necrosis (RIPK-dependent necroptosis) and the networks show that apoptosis as well as necroptosis take place in granulosa cells of the preovulatory luteinising follicles as well as theca of newly selected follicles.

## Figures and Tables

**Table 1:** Assignment of data to respective groups. Developmental stage: A is a newly selected follicle; B is a follicle undergoing differentiation; and C is a follicle undergoing luteinisation.

Granulosa Group			Theca Group		
A1	B1	C1	A2	B2	C2
GCA; GHC	GCB; GHB	GCC; GHC	TCA; THA	TCB; THB	TCC; THC

## Data Availability

None of the data were deposited in an official repository.

## Abbreviations

PCA	Principal Component Analysis
PPI	Protein-Protein Interaction
CL	corpus luteum
GC	granulosa cells
TC	theca cells

## Appendix A

**Table A1:** Uniprot symbols and corresponding gene names used for creating IMEX network.

Uniprot Symbol	Gene Name	Uniprot Symbol	Gene Name	Uniprot Symbol	Gene Name
Q13489	BIRC3	A0A087WTM9	FAS	P12830	CDH1
Q9BRQ8	AIFM2	Q9H1Y0	ATG5	Q8IX12	CCAR1
Q14790	CASP8	P19438	TNFRSF1A	O94768	STK17B
Q9NQC7	CYLD	Q07812	BAX	Q96KQ4	PPP1R13B
P03372	ESR1	Q9UPY3	DICER1	Q9Y275	TNFSF13B
Q15628	TRADD	Q9ULZ3	PYCARD	Q8N474	SFRP1
O00220	TNFRSF10A	O14737	PDCD5	P42575	CASP2
P35869	AHR	P19838	NFKB1	P46527	CDKN1B
P29350	PTPN6	Q9UBF6	RNF7	P48023	FASLG
Q06136	KDSR	P50591	TNFSF10	Q96CV9	OPTN
P55060	CSE1L	P48436	SOX9	Q9UJA3	MCM8
Q9Y572	RIPK3	P55211	CASP9	O15119	TBX3
Q9H0E3	SAP130	Q13546	RIPK1	P29466	CASP1
P11511	CYP19A1	Q9NQX1	PRDM5	P55212	CASP6
Q92731	ESR2	O60936	NOL3	P04637	TP53
O95382	MAP3K6	Q13625	TP53BP2	Q96HF1	SFRP2
O95379	TNFAIP8	O75509	TNFRSF21	P49675	STAR
O95831	AIFM1	P10721	KIT	Q6P589	TNFAIP8L2
Q9UEE5	STK17A	O15551	CLDN3	P55210	CASP7
Q9ULY5	CLEC4E	P35222	CTNNB1	Q13158	FADD
Q8NB16	MLKL	Q9NQS1	AVEN	Q8WUM4	PDCD6IP
P10415	BCL2	Q9UMX3	BOK	Q96IZ0	PAWR
Q6ZN16	MAP3K15	Q6ZMQ8	AATK	Q5GJ75	TNFAIP8L3
P58012	FOXL2	O15304	SIVA1	Q03169	TNFAIP2
P49662	CASP4	Q99683	MAP3K5	P14060	HSD3B1
Q19T08	ECSCR				

**Table A2:** Genes with the highest degree (number of edges) (selected from the 75 genes listed in Table A1).

Uniprot Symbol	Gene Name	Degree
Q14790	CASP8	16
Q13158	FADD	14
Q13546	RIPK1	12
Q94572	Tubulin alpha-3 chain	9
P35222	CTNNB1	8
P04637	TP53	8
P03372	ESR1	7
Q15628	TRADD	7
Q13489	BIRC3	6
O00220	TNFRSF10A	6
Q13625	TP53BP2	6
P10415	BCL2	6
P19438	TNFRSF1A	6
Q92731	ESR2	5
Q9UL23	VEGFA	5

**Table A3:** Genes with the highest betweenness centrality (selected from the 75 genes listed in Table A1).

Uniprot Symbol	Gene Name	Betweenness Centrality Score
Q14790	CASP8	491
Q13158	FADD	435
P35222	CTNNB1	311
P55060	CSE1L	306
P04637	TP53	295
Q13489	BIRC3	248
P19838	NFKB1	227
Q9Y275	TNFSF13B	208
Q13625	TP53BP2	200
P03372	ESR1	187
Q92731	ESR2	150
Q13546	RIPK1	123
Q94572	Tubulin alpha-3 chain	98
P19438	TNFRSF1A	93
P55210	CASP7	78

**Table A4:** Down-regulated genes between subgroups at the False Discovery Rate (FDR) of 0.1.

A1 vs. A2	A1 vs. B1	A1 vs. C1	A2 vs. B2	A2 vs. C1	A2 vs. C2	B1 vs. C1	C1 vs. C2	B2 vs. C2
TNFRSF1A	HSD3B1	KIT	STAR	CYLD	KIT	KIT	CASP4	KIT
CASP8	TNFRSF1A	CYLD	AIFM1	KIT	FAS	CYLD	TNFRSF1A	FAS
CASP4	STK17B	TNFRSF1A	MAP3K5	STK17B	STK17B	BOK	CASP8	STK17B
BOK		STK17B	TMBIM4	MAP3K5	NOL3	STK17B	RIPK3	RIPK1
CASP7		TP53	AIFM2	TP53	CASP6	CASP8	STAR	PYCARD
TRADD		BOK		AIFM2	MAP3K5	TP53	PYCARD	CYLD
STAR		RIPK1		KDSR	CYLD	CASP7	ESR2	ESR2
AHR		HSD3B1		HSD3B1	KDSR	RIPK1	CFLAR	NOL3
RIPK3		CASP7		GATA4		AHR	FAS	CASP8
RIPK1		CASP8		FAS		TNFRSF1A	BIRC3	
KIT		TRADD		AIFM1		AIFM2	CASP7	
PPP1R13B		AHR		NOL3		TRADD	FOXL2	MLKL
TP53		AIFM2		MCM8		CASP4	PPP1R13B	
CFLAR		GATA4				CFLAR		
HSD3B1		STAR						
PYCARD		STAR						

**Table A5:** Up-regulated genes between subgroups at the False Discovery Rate (FDR) of 0.1.

A1 vs. A2	A1 vs. B1	A1 vs. C1	A2 vs. B2	A2 vs. C1	A2 vs. C2	B1 vs. C1	C1 vs. C2	B2 vs. C2
ESR2	CFLAR	ESR2	CASP6	CASP4	FOXL2	ESR2	CYLD	AIFM1
MAP3K5	BOK	FOXL2	TRADD	FOXL2	PPP1R13B	FOXL2	STK17B	FOXL2
AIFM1	RNF7	AIFM1	MLKL	CASP8	TP53	AIFM1	TP53	STAR
RNF7	PYCARD	BIRC3		RIPK3	TRADD	BIRC3	MAP3K5	PPP1R13B
KDSR	CASP8	RNF7		PPP1R13B	AIFM1	NOL3	AIFM1	TMBIM4
FAS		TMBIM4		TNFRSF1A	BIRC3	CCAR1	KIT	TP53
FOXL2		FAS		CFLAR	RNF7	RNF7	GATA4	FADD
NOL3		CCAR1		BIRC3		TMBIM4	AIFM2	
MCM8		NOL3		STAR		MAP3K5	HSD3B1	
CCAR1		MAP3K5		PYCARD		MCM8	CASP6	
TMBIM4		MCM8		ESR2		PPP1R13B	KDSR	
CASP3		CFLAR		AHR		KDSR	FADD	
BIRC3		PPP1R13B		TRADD		FAS	CCAR1	
CYLD		KDSR		CASP7			RNF7	
		CASP3						

FDR value was chosen as a compromise between good sensitivity without an excessive level of FDR, although of course there will be false discoveries.

**Table A6:** The list of genes’ transcripts used to create the CluePedia networks for the respective groups.

A1	B1	C1	A2
TP53-201	TP53-201	TP53-201	TP53-201
AIFM2-202	AIFM2-202	AIFM2-202	AIFM2-202
RNF7-201	RNF7-201	RNF7-201	RNF7-201
NAIP-201	NAIP-201	NAIP-201	NAIP-201
PPP1R13B-202	PPP1R13B-202	PPP1R13B-202	PPP1R13B-202
TNFRSF1A-201	TNFRSF1A-201	TNFRSF1A-201	TNFRSF1A-201
ESR2-201	ESR2-201	ESR2-201	ESR2-201
GATA4-201	GATA4-201	GATA4-201	GATA4-201
TMBIM4-201	TMBIM4-201	TMBIM4-201	TMBIM4-201
CYLD-201	CYLD-201	CYLD-201	CYLD-201
RIPK1-201	RIPK1-201	RIPK1-201	RIPK1-201
CASP7-201	CASP7-201	CASP7-201	CASP7-201
AIFM1-201	AIFM1-201	AIFM1-201	AIFM1-201
KDSR-201	KDSR-201	KDSR-201	KDSR-201
AHR-201	AHR-201	AHR-201	AHR-201
STK17A-201	STK17A-201	STK17A-201	STK17A-201
BOK-201	HSD3B1-201	BOK-201	HSD3B1-201
MAP3K5-201	BOK-201	MAP3K5-201	BOK-201
FAS-201	MAP3K5-201	FAS-201	MAP3K5-201
CFLAR-205	FAS-201	CFLAR-205	FAS-201
XAF1-204	CFLAR-205	XAF1-204	CFLAR-205
CASP6-201	XAF1-204	CASP6-201	XAF1-204
TRADD-201	CASP6-201	TRADD-201	CASP6-201
STK17B-201	TRADD-201	STK17B-201	TRADD
BAX	STK17B-201	BAX	STK17B-201
MCM8-201	BAX	MCM8-201	BAX
CASP3-201	MCM8-201	CYP19A1-201	MCM8-201
CASP2-201	CYP19A1-201	CASP3-201	CYP19A1-201
FADD-201	CASP3-201	CASP2-201	CASP3-201
RIPK3-202	CASP2-201	FADD-201	CASP2-201
PYCARD-201	FADD-201	RIPK3-202	FADD-201
CASP4-201	RIPK3-202	BCL2-201	RIPK3-202
BIRC3-201	PYCARD-201	PYCARD-201	BCL2-201
CASP8-202	CASP4-201	CASP4-201	PYCARD-201
FOXL2-201	BIRC3-201	BIRC3-201	CASP4-201
STAR-201	CASP8-202	CASP8-202	BIRC3-201
CCAR1-202	FOXL2-201	FOXL2-201	TNF-201
MLKL	STAR-201	STAR-201	CASP8-202
	CCAR1-202	CCAR1-202	FOXL2-201
	MLKL	MLKL	STAR-201
			CCAR1-202
			MLKL

**Table A7:** Characteristics of real-time PCR.

Gene Symbol	GenBank Accession No.	Primer Size (bp)	Amplicon Size (bp)	Forward Primer / Reverse Primer (bp)
GAPDH	NM_001035012.1	20	103	5’CACCCTCAAGATTGTCAGCA’3
				5’GGTCATAAGTCCCTCCACGA’3
RIPK1	NM_001035012.1	20	148	5’GCAATAGCTCCAAGCAGGTC’3
				5’TGTGCAGCAGGAAGTCATTC’3
RIPK3	NM_001101884.2	20	219	5’CCAGAGAGCAGGTTCCAC’3
				5’AATCAGGCGGTTGTTGTTTC’3

**Table A8:** The list of genes used in PCA and Up- and Down-regulation analyses.

Uniprot Symbol	Gene Name
P67939	TP53
A5PJM4	AIFM2
Q0P5F4	RNF7
A0A452DHX3	KIT
F1MVS4	NOL3
E1BHY2	PPP1R13B
F1MSX5	TNFRSF1A
Q9XSB5	ESR2
F1N551	GATA4
Q58DU1	CGI-119
Q1RMU2	CYLD
Q3SZK6	RIPK1
F1MD58	CASP7
E1BJA2	AIFM1
F1MLE5	KDSR
F1ML85	AHR
A0A452DIY6	HSD3B1
A6H6W7	BOK
F1MXH6	MAP3K5
A5D7R0	FAS
A0A3S5ZPV3	CFLAR
G3X702	CASP6
Q2KI74	TRADD
F1MSI1	STK17B
E1BPX4	MCM8
F1MB04	CASP3
E1BJT6	CASP2
Q645M6	FADD
F1MQF6	PYCARD
Q5E9C1	CASP4
Q3SYW8	BIRC3
Q2LGB8	CASP8
Q6VFT7	FOXL2
Q28918	STAR
E1BJ81	CCAR1
E1BKA8	RIPK3
